# Magnetic-Field-Tunable High-Resistivity State Strongly Coupled to Complex Magnetism in NdSb_0.5_Te_1.5_

**DOI:** 10.3390/ma19214739

**Published:** 2026-11-08

**Authors:** Minju Jeong, Igor Plokhikh, Ekaterina Pomjakushina, Jongtae Ahn, Soonbeom Seo

**Affiliations:** 1 Department of Physics, Changwon National University, Changwon 51140, Republic of Korea; minju051800@gmail.com (M.J.); jongtae@changwon.ac.kr (J.A.); 2 PSI Center for Neutron and Muon Sciences, Paul Scherrer Institut, 5232 Villigen, Switzerland; igor.plokhikh@tu-dortmund.de (I.P.); ekaterina.pomjakushina@psi.ch (E.P.)

**Keywords:** topological material, high resistivity state, magnetoresistance, NdSbTe

## Abstract

NdSbTe is a magnetic topological semimetal in which nontrivial electronic states coexist with antiferromagnetic order, while variation in the Sb/Te composition substantially modifies its electronic and magnetic properties. Here, we investigate the temperature- and magnetic field-dependent electrical transport properties of NdSb_0.5_Te_1.5_. The electrical resistivity exhibits nonmetallic behavior below approximately 100 K and develops a pronounced high-resistivity state below 4 K, reaching more than three orders of magnitude above its room temperature value. Multiple characteristic anomalies emerge within the high-resistivity state and are systematically suppressed by magnetic fields. The characteristic temperatures and critical fields consistently define multiple transport regimes in the H-T phase diagram. Remarkably, a relatively weak magnetic field of approximately 0.5 T induces an abrupt and hysteretic transition between high and low resistivity states. These results demonstrate an exceptionally large field-tunable resistive response closely coupled to the magnetic state in NdSb_0.5_Te_1.5_.

## 1. Introduction

The interplay between magnetism and nontrivial electronic topology provides a fertile ground for understanding unconventional quantum states and transport phenomena [1,2,3,4]. In magnetic topological materials, changes in magnetic order modify the underlying electronic states, providing a route to control electrical transport through external parameters such as magnetic fields [2,5]. Materials in which magnetism, electronic topology, and charge transport coexist are therefore of considerable interest for exploring coupled electronic and magnetic states. More broadly, strong coupling between magnetic and electronic states can produce large field-tunable resistive responses, as demonstrated in mixed-valence manganites where competing magnetic interactions, carrier localization, and charge ordering give rise to pronounced magnetoresistance [6].

The LnSbTe (Ln = lanthanide) family provides an attractive platform for such studies. These compounds consist of Sb square nets separated by Ln-Te layers, where topological electronic states associated with the square net coexist with localized 4*f* magnetic moments [7,8,9,10]. Stoichiometric NdSbTe is an antiferromagnetic topological semimetal with a magnetic ordering temperature near 3 K and a relatively large Sommerfeld coefficient of γ~115 mJ/mol K^2^, suggesting enhanced electronic correlations possibly associated with the hybridization between localized 4*f*-electrons and conduction electrons [9,11]. In addition, angle-resolved photoemission spectroscopy and DFT calculations have revealed multiple gapless nodal-line states, demonstrating the nontrivial electronic topology of NdSbTe [12].

In NdSb_x_Te_2−x−δ_, decreasing the Sb content results in partial substitution of Sb by Te within the square-net layer, providing a means of tuning both the electronic and magnetic properties [13,14]. Such substitution changes the band filling of the square net and induces structural distortions associated with charge-density-wave (CDW) formation [14,15]. In the intermediate-composition region, the tetragonal symmetry (P4/nmm) of stoichiometric NdSbTe is reduced, and the magnetic ground state evolves substantially with composition, from the relatively simple antiferromagnetic order of nearly stoichiometric NdSbTe to more complex magnetic structures [13,14]. For instance, in a composition close to NdSb_0.5_Te_1.5_, neutron diffraction revealed an incommensurate elliptical cycloidal magnetic structure, together with a relationship between the magnetic and structural modulation vectors [14]. These observations demonstrate that compositional variation strongly modifies both the electronic environment and magnetic ground state of NdSb_x_Te_2−x−δ_. Although a recent transport study reported a systematic evolution of magnetoresistance with Sb/Te composition in NdSb_x_Te_2−x_ [15], the detailed electrical transport response associated with the complex magnetic states in the intermediate-composition region remains less explored. In particular, the low-temperature transport anomalies associated with the phase transitions and their detailed evolution under magnetic fields have not been systematically investigated.

In this study, we investigate the temperature- and magnetic-field-dependent electrical transport of NdSb_0.5_Te_1.5_. We observe an exceptionally large low-temperature resistive response that is strongly modified by a relatively weak magnetic field. The resulting H-T phase diagram reveals multiple field-tunable transport regimes, demonstrating a strong coupling between the magnetic state and electrical transport.

## 2. Materials and Methods

The samples were grown by the chemical vapor transport (CVT) method following procedures similar to those previously reported for LnSbTe compounds [9,13,16,17]. The starting materials (Nd Goodfellow 3N, Sb Chempur 5N, Te Chempur 5N) were mixed according to the nominal composition of NdSb_0.5_Te_1.5_ and pre-reacted by annealing at 900 °C for 24 h in an evacuated silica ampule. Ca. 3 g of the thus obtained powder was loaded in an 8 mm-diameter quartz ampule with ca. 20 mg of I2, flame-sealed and placed into a gradient furnace. The transport reaction was performed in the temperature gradient 1000–900 °C, from the hot to the cold zone for ca. 4 weeks. This procedure yields mm-sized single crystals used further for physical property measurements. Single-crystal X-ray diffraction measurements were performed using a STOE STADIVARI diffractometer (STOE & Cie GmbH, Darmstadt, Germanay) with Mo Kα radiation (λ = 0.71047 Å), and powder X-ray diffraction measurements were performed on crushed single crystals using a BRUKER AXS D8 ADVANCE diffractometer (Bruker AXS GmbH, Karlsruhe, Germany) with Cu Kα radiation. The diffraction data were used to characterize the crystal structure and estimate the sample composition, as discussed in Section 3. A standard four-probe method was employed to measure the electrical resistivity, using an AC resistance bridge. To improve electrical contact with the sample surface, Ti (40 nm)/Au (10 nm) electrodes were fabricated via conventional photolithography, e-beam evaporation, and a standard lift-off process. All transport measurements were performed with the magnetic field applied parallel to the *c*-axis and the electrical current flowing within the *ab*-plane. The resistivity and magnetoresistance measurements were carried out in a cryogen-free superconducting magnet system and Quantum Design Physical Property Measurement System (PPMS Dynacool) over the temperature range from 300 K to 1.8 K.

## 3. Results and Discussion

**Figure 1:**
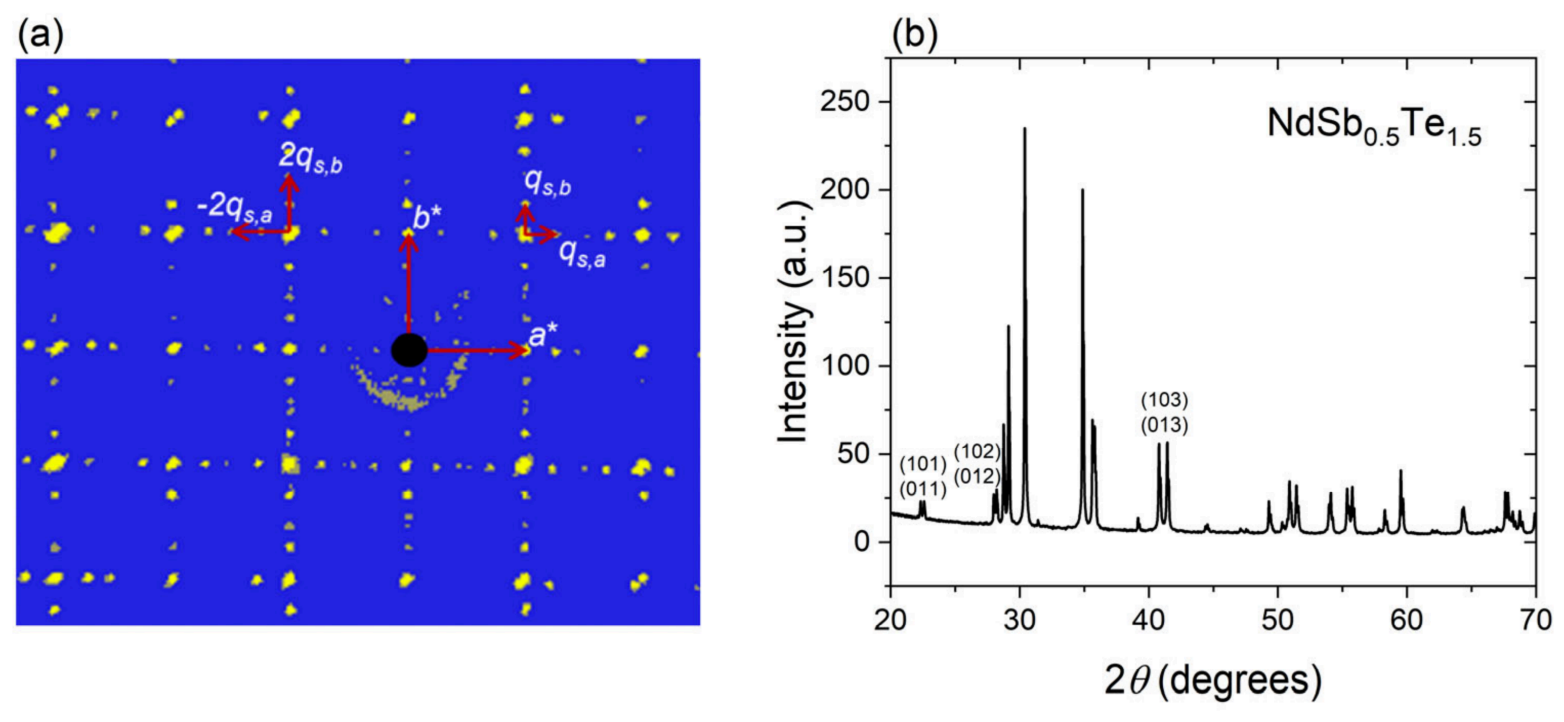
X-ray diffraction characterization of NdSb_0.5_Te_1.5_. (**a**) Reconstructed hk2 reciprocal-space plane obtained from single-crystal X-ray diffraction, showing the principal reciprocal-lattice axes (a*, b*) and first- and second-order incommensurate satellite reflections. (**b**) Room-temperature powder X-ray diffraction pattern showing selected split hkl and khl reflections associated with the orthorhombic distortion.

Figure 1 presents the single-crystal and powder X-ray diffraction results of NdSb_0.5_Te_1.5_. The diffraction data confirm an incommensurately modulated orthorhombic structure with the superspace group *Pmmn*(0β0)00s. The single-crystal diffraction pattern reveals incommensurate satellite reflections with a modulation vector of *q_s_* = (0, 0.2700(4), 0), while the powder diffraction pattern exhibits splitting of the hkl and khl reflections associated with the orthorhombic distortion. The lattice parameters obtained from powder XRD refinement are a = 4.3592(1) Å, b = 4.4247(1) Å, and c = 9.2008(1) Å. Structural refinement indicates approximately 5% vacancies at the mixed Sb/Te site. Because of the similar X-ray scattering factors of Sb and Te, their individual occupancies cannot be reliably determined. Assuming that the nominal Sb/Te ratio is preserved, the composition is estimated to be approximately NdSb_0.49_Te_1.46_, close to the nominal composition of NdSb_0.5_Te_1.5_ used throughout this study.

**Figure 2:**
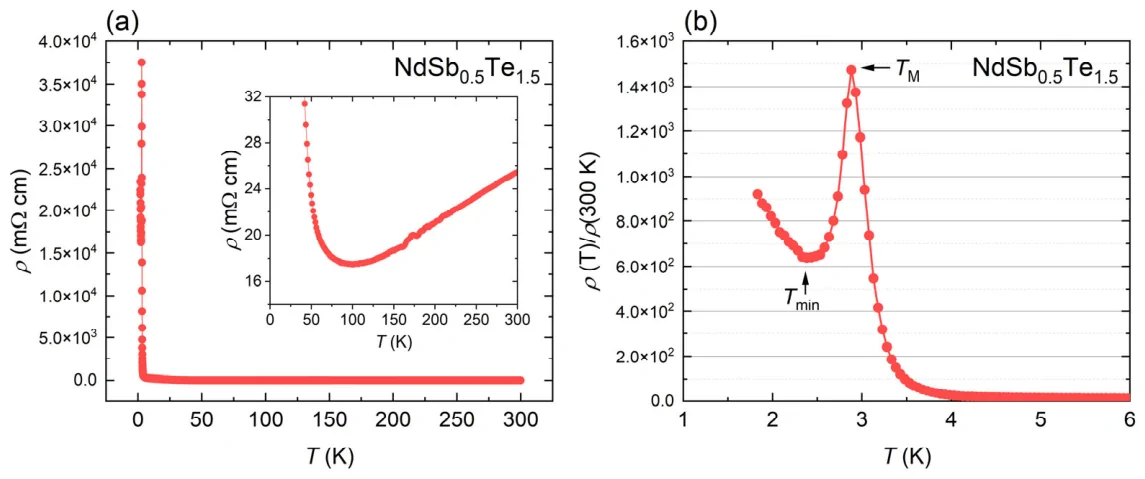
Temperature dependence of the electrical resistivity of NdSb_0.5_Te_1.5_. (**a**) Electrical resistivity, *ρ*(T), measured from 300 to 1.8 K. The inset shows the low-temperature resistivity on an enlarged scale. (**b**) Normalized resistivity, *ρ*(T)/*ρ*(300 K), below 6 K. *T_M_* and *T_min_* indicate the characteristic temperatures corresponding to the sharp resistivity maximum and local minimum, respectively.

Figure 2a shows the temperature dependence of the electrical resistivity, *ρ*(T), of NdSb_0.5_Te_1.5_ from 300 to 1.8 K. The resistivity decreases linearly with decreasing temperature from room temperature to approximately 100 K, exhibiting conventional metallic behavior. Below 100 K, however, *ρ*(T) deviates from the metallic temperature dependence and gradually increases upon further cooling, as clearly shown in the inset of Figure 2a. Although nonmetallic transport behavior has also been reported in stoichiometric NdSbTe and other magnetic LnSbTe compounds [11,18,19,20], NdSb_0.5_Te_1.5_ exhibits a remarkably stronger enhancement of resistivity at lower temperatures. Figure 2b shows the normalized resistivity, *ρ*(T)/*ρ*(300 K), below 6 K. The resistivity begins to increase rapidly below approximately 4 K and exhibits a sharp peak-like anomaly at *T_M_* = 2.84 K, where the resistivity becomes more than three orders of magnitude larger than its room-temperature value. Although a peak-like resistive anomaly near the antiferromagnetic ordering temperature has also been reported in stoichiometric NdSbTe [11], the enhancement observed in NdSb_0.5_Te_1.5_ is substantially larger. This pronounced difference suggests that the modification of the Sb/Te composition strongly alters not only the magnetic ground state but also the electronic states responsible for charge transport. With further cooling below *T_M_*, the resistivity rapidly decreases and reaches a local minimum at *T_min_* = 2.37 K, followed by another upturn toward the lowest measured temperature. The nonmonotonic evolution of *ρ*(T) suggests the development of an additional low-temperature state below *T_min_*.

**Figure 3:**
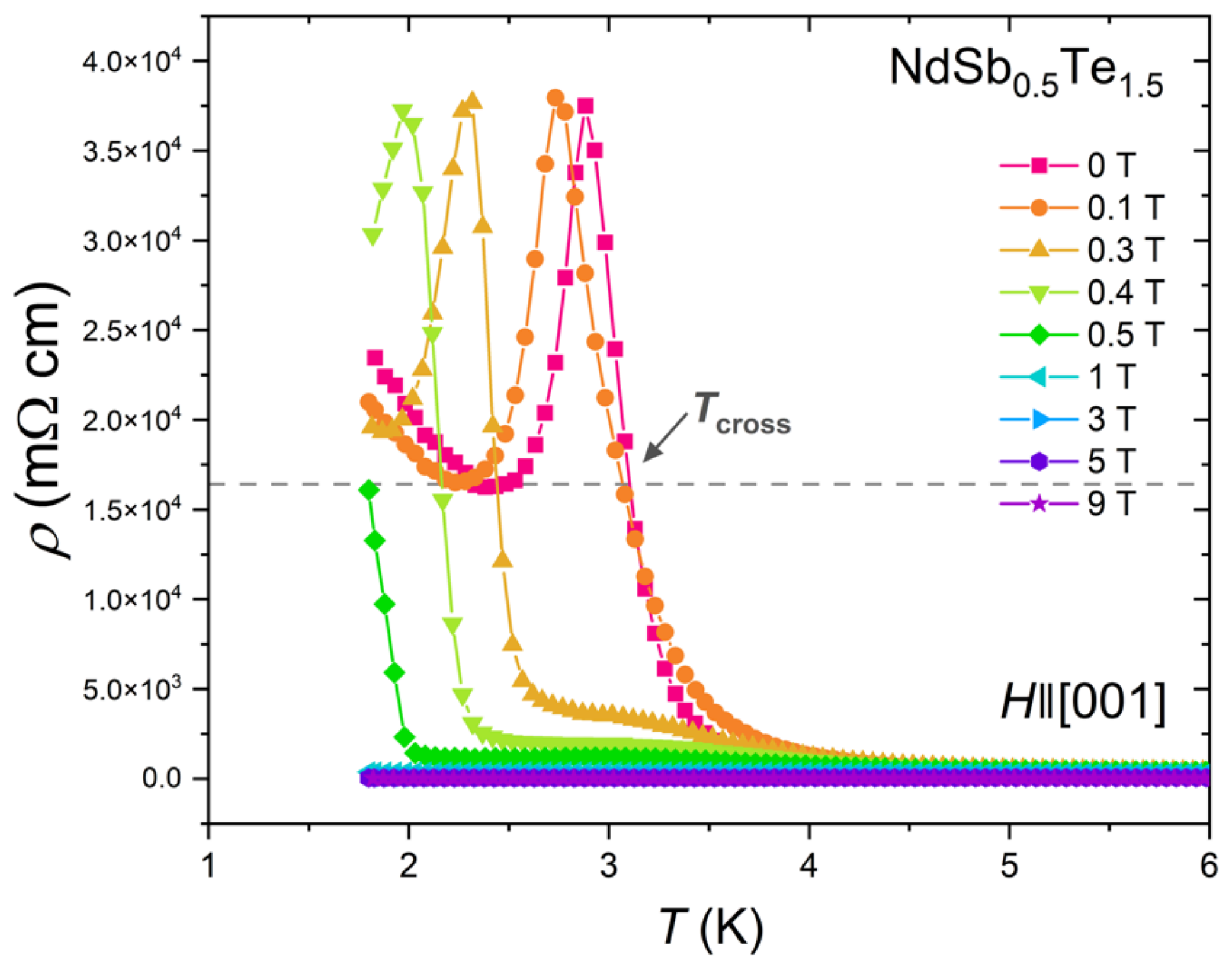
Temperature dependence of the electrical resistivity of NdSb_0.5_Te_1.5_ under magnetic fields applied along the *c*-axis. *T_cross_* is defined as the temperature at which the resistivity reaches half of the increase from its value at 4 K to the maximum resistivity.

Figure 3 shows the temperature dependence of the electrical resistivity under magnetic fields up to 9 T applied along the *c*-axis. With increasing magnetic field, both *T_M_* and *T_min_* are continuously shifted toward lower temperatures. In addition, the onset of the pronounced resistive upturn is systematically shifted to lower temperatures. To characterize this field evolution, we define *T_cross_* as the temperature at which the resistivity reaches half of the increase from its value at 4 K to the maximum resistivity. *T_cross_* is also continuously suppressed with increasing magnetic field. The pronounced resistive upturn remains observable up to 0.5 T, whereas it is no longer observed above 1 T within the measured temperature range. The simultaneous suppression of *T_cross_*, *T_M_*, and *T_min_* by relatively weak magnetic fields indicates that these characteristic transport anomalies are closely associated with the magnetic state of NdSb_0.5_Te_1.5_. This behavior is consistent with previous studies of NdSb_x_Te_2−x−δ_, in which the magnetic states in the intermediate-composition region were found to be highly sensitive to applied magnetic fields [13,14]. The similar field scale for suppressing the transport anomalies therefore suggests a strong coupling between the magnetic state and electronic transport in NdSb_0.5_Te_1.5_.

**Figure 4:**
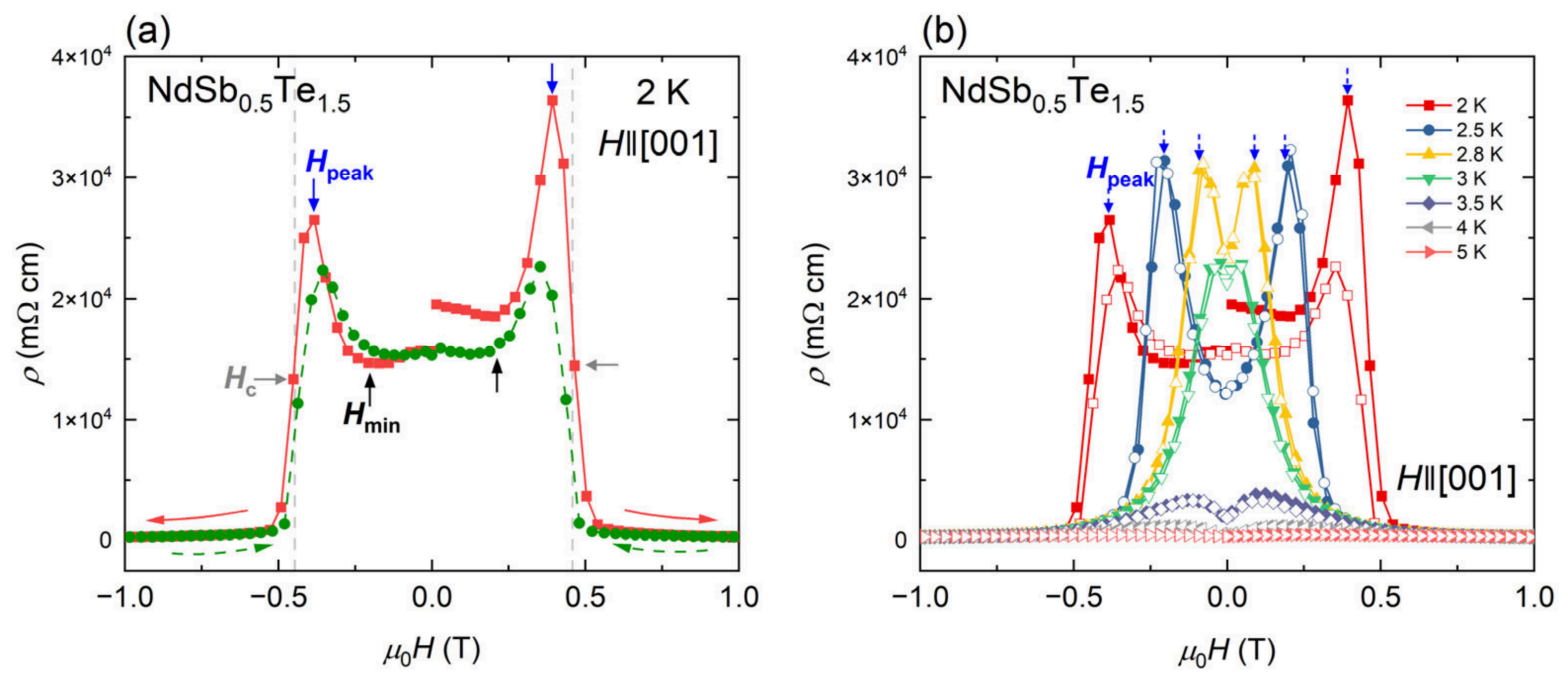
Magnetic-field dependence of the electrical resistivity of NdSb_0.5_Te_1.5_ for *H*//*c*-axis. (**a**) *ρ*(H) measured at 2 K between −1 and 1 T. Arrows indicate the magnetic-field sweep directions. *H_min_*, *H_peak_*, and *H_c_* denote the characteristic fields corresponding to the local resistivity minimum, resistivity maximum, and midpoint of the abrupt resistive transition, respectively. (**b**) *ρ*(H) measured at various temperatures, showing the temperature evolution of the characteristic field scales.

Figure 4a shows the magnetic-field dependence of the electrical resistivity, *ρ*(H), at 2 K for magnetic fields applied along the *c*-axis. Starting from zero field, the resistivity initially decreases with increasing field and reaches a local minimum at approximately 0.25 T, which is defined as *H_min_*. With further increasing field, the resistivity rises again and exhibits a pronounced maximum at *H_peak_*, followed by an abrupt decrease at a slightly higher magnetic field. We define *H_c_* as the midpoint of this sharp resistive drop. Above approximately 0.5 T, the system remains in a low-resistivity state with only a weak field dependence. Pronounced hysteretic behavior is observed around *H_peak_* and *H_c_* between increasing- and decreasing-field sweeps. The hysteresis indicates that the abrupt resistive change is unlikely to originate from a simple continuous magnetoresistive response and instead suggests a first-order-like field-induced transition. The sharp and hysteretic collapse of the high-resistivity state therefore provides further evidence that the unusual electrical transport is intimately associated with the underlying magnetic state. Figure 4b presents *ρ*(H) measured at various temperatures. The characteristic features observed at 2 K evolve systematically with increasing temperature. The *H_min_* anomaly is observed only at the lowest temperatures and disappears above 2.5 K. Meanwhile, both *H_peak_* and *H_c_* progressively shift toward lower magnetic fields with increasing temperature. Despite the substantial shift in these characteristic fields, the abrupt change in resistivity across *H_c_* remains remarkably large at low temperatures, approaching three orders of magnitude. Above approximately 3 K, the sharp H_peak_ anomaly disappears, and the magnitude of the field-induced resistivity variation is strongly reduced. At higher temperatures, the magnetoresistive response becomes progressively weaker, and above approximately 5 K, no pronounced field-induced resistive anomaly is observed. The distinct temperature evolutions of *H_min_*, *H_peak_*, and *H_c_*, together with the hysteretic behavior, demonstrate the presence of multiple field-tunable transport regimes. Remarkably, a magnetic field of less than approximately 0.5 T is sufficient to switch the system between states with drastically different electrical resistivities, indicating a strong coupling between the magnetic and electronic states.

**Figure 5:**
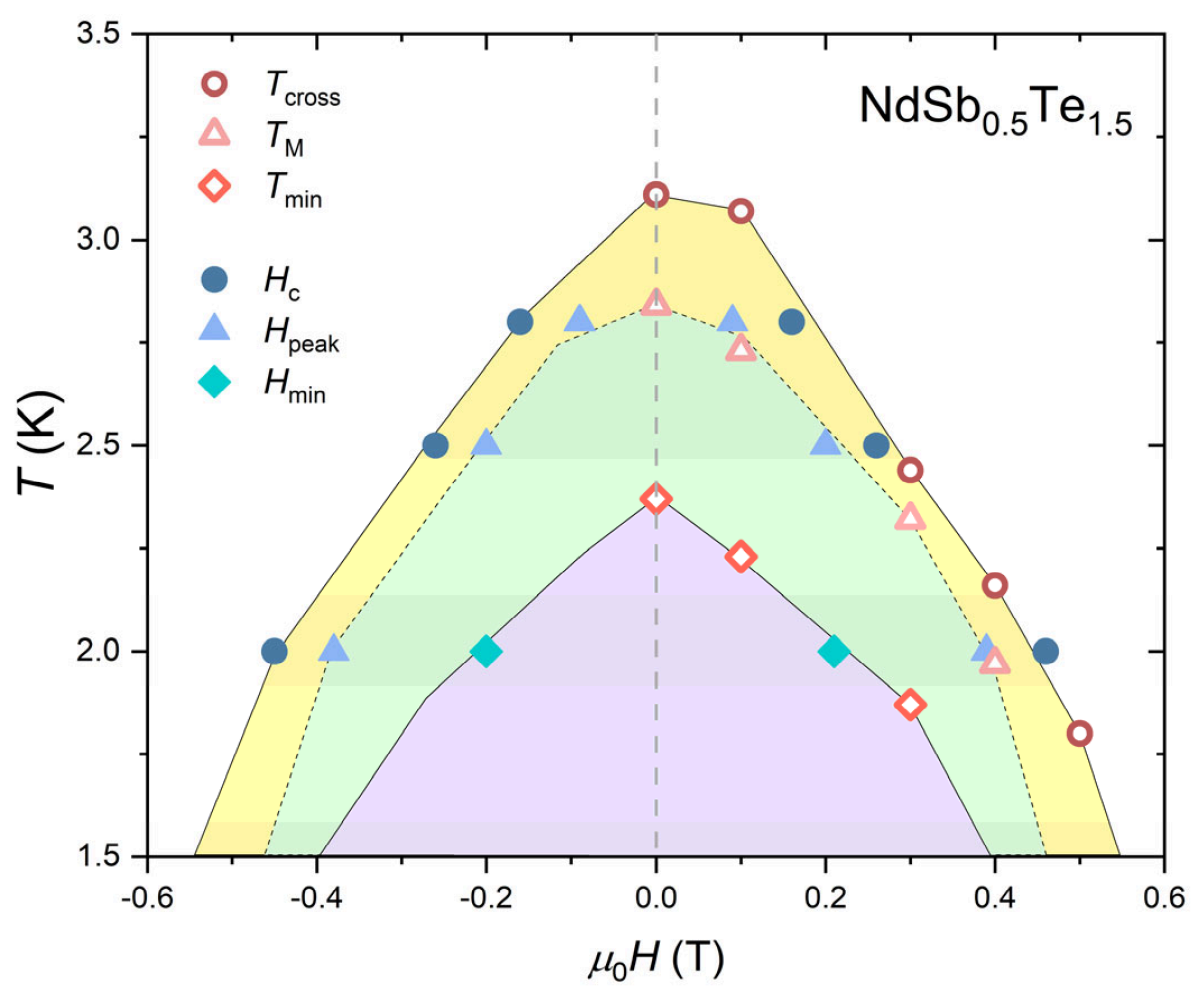
Magnetic field–temperature (H-T) phase diagram of NdSb_0.5_Te_1.5_ constructed from the electrical transport measurements. Open symbols represent the characteristic temperatures *T_cross_*, *T_M_*, and *T_min_* determined from *ρ*(T), while solid symbols represent the corresponding characteristic fields *H_c_*, *H_peak_*, and *H_min_* determined from *ρ*(H). The characteristic temperature and field scales define the boundaries between the different transport regimes.

Figure 5 summarizes the magnetic field–temperature (H-T) phase diagram of NdSb_0.5_Te_1.5_ constructed from the characteristic temperatures and fields determined from the electrical transport measurements. The open symbols represent the characteristic temperatures extracted from *ρ*(T), whereas the solid symbols denote the characteristic fields obtained from *ρ*(H). Remarkably, the independently determined temperature and field scales show a systematic correspondence: *T_cross_* and *H_c_*, *T_M_* and *H_peak_*, and *T_min_* and *H_min_* continuously connect to form three distinct boundaries in the H-T phase diagram. The outer boundary defined by *T_cross_* and *H_c_* encloses the high-resistivity state observed at low temperatures and low magnetic fields. Their continuous evolution indicates that the high-resistivity state is suppressed by either increasing temperature or magnetic field. Within this region, the *T_M_* and *H_peak_* anomalies define an additional transport boundary. Considering the antiferromagnetic ordering reported in previous studies of closely related compositions [13,14], this boundary is likely associated with the evolution of the magnetically ordered state. Neutron diffraction measurements on the closely related NdSb_0.48_Te_1.37_ compound revealed an incommensurate elliptical cycloidal magnetic structure [14]. More intriguingly, *T_min_* and *H_min_* form an additional boundary entirely within the magnetically ordered region. The close correspondence between *T_min_*, determined from *ρ*(T), and *H_min_*, independently determined from *ρ*(H), suggests that this feature is not simply a resistive crossover but may represent an additional low-temperature state, possibly associated with a modification or reconstruction of the magnetic ground state. We emphasize that all boundaries in the present H–T diagram are defined solely from electrical transport measurements. Therefore, although their systematic correspondence and comparison with previous magnetic studies suggest a close relationship with the magnetic state, direct magnetic measurements will be required to determine the magnetic structures associated with these transport regimes.

The present results further demonstrate that changing the Sb/Te composition substantially modifies the electronic transport characteristics of NdSb_x_Te_2−x−δ_. While the previous transport study [15] focused on the systematic evolution of magnetoresistance across the NdSb_x_Te_2−x_ series, the present work provides a detailed investigation of the low-temperature transport behavior of the intermediate composition NdSb_0.5_Te_1.5_. In particular, our measurements reveal the development of an exceptionally high-resistivity state and its abrupt and hysteretic transition to a low-resistivity state under a relatively weak magnetic field. Stoichiometric NdSbTe hosts topological semimetallic electronic states associated with its square-net structure, whereas NdSb_0.5_Te_1.5_ exhibits strongly nonmetallic low-temperature transport and develops an exceptionally high-resistivity state. The resistivity reaches more than three orders of magnitude above its room-temperature value and can subsequently be suppressed by a magnetic field of approximately 0.5 T. This dramatic change indicates a substantial reconstruction of the electronic states responsible for charge transport upon changing the Sb/Te composition. One possible origin of this unusual transport behavior is the composition-induced reconstruction of the Sb/Te square net associated with charge-density-wave (CDW) formation. Previous studies of NdSb_x_Te_2−x−δ_ have demonstrated that varying the Sb content changes the band filling of the square net and produces structural distortions accompanied by an incommensurate CDW [14]. In a composition close to NdSb_0.5_Te_1.5_, the CDW substantially reconstructs the square net, while neutron diffraction further demonstrates a close relationship between the CDW modulation and the incommensurate magnetic structure. In particular, the magnetic and CDW propagation vectors exhibit an integer relationship, suggesting that the electronic reconstruction of the square net is directly coupled to the magnetic ordering through conduction-electron-mediated interactions [14]. Such a CDW-induced reconstruction may provide a possible framework for understanding the enhanced resistivity and its close connection to the transport-defined boundaries that are likely associated with the evolution of the magnetic state [5,21].

An additional intriguing possibility is that the high-resistivity state is related to a reconstruction of the topological electronic states. Stoichiometric NdSbTe has been experimentally shown to host multiple gapless nodal-line states. Previous studies of related LnSb_x_Te_2−x_ compounds further indicate that structural distortions can substantially reconstruct the electronic states while retaining nontrivial topology in portions of the electronic structure [5,21]. If nontrivial band topology remains preserved in NdSb_0.5_Te_1.5_ while the bulk electronic states become strongly gapped or localized by the composition-induced reconstruction, the high-resistivity state observed here may represent a possible topological-insulator-like electronic state. In SmB_6_, a proposed topological Kondo insulator, strong electronic correlations produce a bulk Kondo hybridization gap, while metallic surface states persist within the insulating bulk gap [22,23,24,25]. The formation of the bulk gap results in a pronounced enhancement of resistivity upon cooling, with surface conduction becoming increasingly important at low temperatures. The microscopic mechanism responsible for the high-resistivity state in NdSb_0.5_Te_1.5_ is likely different from that in SmB_6_; nevertheless, this comparison illustrates how a reconstruction of low-energy electronic states in a correlated topological material can produce a crossover from metallic to highly resistive transport. In SmB_6_, external pressure suppresses the correlation-driven Kondo hybridization gap, thereby driving the system out of the high-resistivity state [26,27]. In contrast, in NdSb_0.5_Te_1.5_, application of a relatively weak magnetic field continuously suppresses *T_cross_* and ultimately induces an abrupt, hysteretic collapse of the high-resistivity state across *H_c_*. Moreover, the magnitude of the high resistivity remains substantial even as the characteristic temperatures and fields are progressively suppressed. These observations indicate that the unusual electronic state is intimately linked to the evolution of the magnetic ground state rather than representing a conventional field-independent insulating behavior. The present transport measurements, however, cannot establish the topological nature of this state.

Even without assigning a specific topological origin, the experimentally observed switching between high- and low-resistivity states demonstrates an exceptionally strong magnetotransport response and highlights NdSb_0.5_Te_1.5_ as a promising platform for field-tunable electronic functionality. Further microscopic investigations are required to establish the origin of the observed transport states. Neutron diffraction below *T_cross_* and *T_min_* will be particularly important for determining whether the additional transport boundaries correspond to changes in the magnetic structure. Electronic-structure calculations, together with spectroscopic measurements such as ARPES, will also be necessary to determine whether nontrivial topological electronic states survive in the high-resistivity regime.

## 4. Conclusions

In summary, we investigated the temperature and magnetic field-dependent electrical transport properties of NdSb_0.5_Te_1.5_. A pronounced high-resistivity state develops below approximately 4 K, accompanied by multiple characteristic anomalies that continuously connect with the corresponding characteristic fields to construct multiple transport regimes in the H-T phase diagram. Remarkably, a relatively weak magnetic field of approximately 0.5 T induces abrupt and hysteretic switching between high and low-resistivity states, demonstrating a strong coupling between the magnetic state and electrical transport. The exceptionally large and field-tunable resistive response suggests that NdSb_0.5_Te_1.5_ provides a promising platform for exploring coupled magnetic and electronic states.

## Data Availability

The original contributions presented in this study are included in the article. Further inquiries can be directed to the corresponding author.
